# Determinants of Postpartum Sexual Dysfunction in the First Year: A Systematic Review

**DOI:** 10.3390/healthcare13222977

**Published:** 2025-11-19

**Authors:** Aris Boarta, Adrian Gluhovschi, Marius Lucian Craina, Carmen Ioana Marta, Bogdan Dumitriu, Ioana Denisa Socol, Madalina Ioana Sorop, Bogdan Sorop

**Affiliations:** 1 Faculty of Medicine, Doctoral School, Victor Babes University of Medicine and Pharmacy, 300041 Timisoara, Romania; aris.boarta@umft.ro (A.B.); bogdan.dumitriu@umft.ro (B.D.); ioana.socol@umft.ro (I.D.S.); madalina.pop@umft.ro (M.I.S.); 2 Department of Obstetrics and Gynecology, Victor Babes University of Medicine and Pharmacy, 300041 Timisoara, Romania; mariuscraina@umft.ro (M.L.C.); carmen.marta@umft.ro (C.I.M.); bogdan.sorop@umft.ro (B.S.)

**Keywords:** postpartum sexual dysfunction, dyspareunia, breastfeeding, partner support, body image, perineal trauma, pelvic pain, FSFI

## Abstract

**Background and Objectives:** This systematic review synthesized somatic and psychosocial determinants of postpartum sexual dysfunction (PSD) during the first 12 months after childbirth. **Methods:** Following PRISMA 2020 guidelines, we searched PubMed, Web of Science, and Scopus from inception to 4 August 2025 without language limits for the indexed records. Eligible studies enrolled postpartum women (≤12 months) and reported validated sexual outcomes (FSFI/FSFI-6, PISQ-12), dyspareunia, or sexual activity, examining breastfeeding, partner support/body image, perineal trauma/instrumentation, or postpartum perineal/musculoskeletal pain. Two reviewers independently screened and extracted data; risk of bias was assessed with a modified Newcastle–Ottawa Scale. **Results:** Of 1127 records screened, 15 studies were included. Perineal morbidity and early pain consistently tracked with worse sexual outcomes; assisted vaginal birth increased 6-month dyspareunia odds (OR 2.5). Breastfeeding was often associated with lower early sexual function and higher dyspareunia (6-month dyspareunia OR 4.4), with attenuation by 12 months. Higher partner/family support and more positive body image correlated with better FSFI scores. Heterogeneity in timing, measures, and adjustment precluded meta-analysis; results were narratively synthesized. **Conclusions:** Perineal trauma and early pain are dominant risk signals; breastfeeding-related symptoms exert early and context-dependent effects; psychosocial resources are protective.

## 1. Introduction

Postpartum sexual dysfunction (PSD)—encompassing diminished desire, arousal difficulties, lubrication problems, orgasmic disturbance, and dyspareunia—affects a large minority of women across the first postnatal year and follows heterogeneous trajectories [1,2]. Large prospective cohorts report that 20–40% of primiparous women still describe painful intercourse at 6–12 months postpartum, with higher rates earlier after birth and among those with obstetric intervention or perineal morbidity [1,3]. Longitudinal analyses also show that while sexual function tends to improve between ~3 and 12 months postpartum, a meaningful subset continues to experience distressing symptoms that impair quality of life and relationships, underscoring the need to identify modifiable determinants and windows for intervention [2,4].

Measurement choices strongly influence reported prevalence and effect sizes. The Female Sexual Function Index (FSFI) and its short form (FSFI-6) are the most frequently used instruments, but they capture overlapping yet distinct domains, and cut-points validated in non-postpartum populations can misclassify transient and normative recovery patterns after birth [5]. Few studies validate postpartum-specific cut-offs for FSFI/FSFI-6, applying general-population thresholds risks misclassifying normative early recovery patterns [6,7].

Biology plausibly links lactation to sexual symptoms via relative hypoestrogenism, reduced vaginal lubrication, and vestibulovaginal tissue sensitivity. “Genitourinary syndrome of lactation” has recently been proposed to emphasize the estrogen-deprived milieu in breastfeeding women, paralleling aspects of genitourinary syndrome of menopause, yet with a distinct clinical context and natural history [8]. Importantly, not all lactating women develop dyspareunia, and cross-sectional clinic data indicate that postpartum vulvovaginal atrophy is not uniformly associated with pain during intercourse, suggesting contributions from perineal scarring, lubrication strategies, sexual avoidance, and relational factors [9].

Obstetric perineal trauma remains a major determinant of postpartum dyspareunia. Contemporary reviews and cohort syntheses indicate that obstetric anal sphincter injury (OASI) carries substantial and multi-year sequelae—including pain, sexual dysfunction, and altered future birth plans—with risk gradients by tear severity and assisted vaginal delivery [10,11]. Quality-improvement programs targeting recognition and preventive strategies for OASI reflect this persistent burden and the need for systematic repair, infection prevention, and early rehabilitation [10,11].

Pain phenotypes that persist beyond the early puerperium—particularly perineal pain and pelvic girdle pain (PGP)—interface with sexual function through avoidance learning, fear of pain, reduced arousal, and mood symptoms. Case–control evidence within the first six months postpartum links PGP to differences in pelvic floor parameters and psychosocial factors, supporting a biopsychosocial model and the potential utility of pelvic health assessment and graded exposure [12]. Beyond somatic drivers, dyadic studies demonstrate that depressive symptoms correlate with lower sexual function and higher sexual distress for both members of the couple at three months postpartum, with partial recovery thereafter—suggesting that early screening and stepped-care management of mood may indirectly improve sexual outcomes [13].

Nevertheless, psychosocial context, including partner support and body image, shapes postpartum sexuality but is less frequently foregrounded in syntheses. Prospective and cross-sectional studies show postpartum degradation of body image and its associations with poorer sexual function or reduced sexual frequency; these effects coexist with role transitions, fatigue, and caregiving demands [14]. Qualitative work further situates postpartum sexuality within mothers’ “sexual worldviews,” relational climates, and cultural scripts, underscoring why partner support and body image may be modifiable levers with outsized influence on desire, arousal, and satisfaction [15].

No prior synthesis has disaggregated sexual pain trajectories by perineal tear grade and parity with standardized timepoints, and psychosocial domains (support and body image) are underrepresented alongside somatic factors. We focus on four pre-specified domains. Mental health and postnatal hormonal therapy were considered cross-cutting modifiers and are discussed as limitations of scope rather than primary determinants. We aimed to synthesize evidence on four determinant domains of postpartum sexual dysfunction (PSD)—(i) breastfeeding, (ii) partner support and body image, (iii) perineal trauma/instrumentation, and (iv) postpartum perineal/musculoskeletal pain—during the first 12 months after birth, and to appraise evidence certainty.

## 2. Materials and Methods

### 2.1. Protocol and Eligibility Criteria

The current study followed PRISMA 2020 recommendations [16] (Supplementary File: PRISMA checklist) and was registered with the Open Science Framework with the registration code: osf.io/rj2pe. We included primary studies (cohort, case–control, cross-sectional, and registry-based), enrolling postpartum women (up to 12 months postpartum unless otherwise specified) who reported at least one validated sexual function outcome with FSFI (full version) or FSFI-6, Sexual Activity Questionnaire (SAQ), PISQ-12, or explicit dyspareunia prevalence, and explicitly examined at least one of the following determinants: (a) breastfeeding/infant-feeding status; (b) partner/family support or body image; (c) perineal trauma/instrumentation (including tear grade, episiotomy, vacuum/forceps); (d) postpartum perineal or musculoskeletal pain. We excluded pure intervention trials unless baseline associations were separately analyzable; narrative reviews, meta-analyses, editorials, and qualitative-only studies; studies restricted to pregnancy (without postpartum outcomes); studies lacking a total *n* or any extractable numerical outcome. When multiple timepoints were presented, we extracted the postpartum timepoint closest to 3–12 months.

No language restrictions were applied for PubMed-indexed records. Primary outcomes were (i) presence of sexual dysfunction (per study’s instrument cut-off), (ii) FSFI total/domain scores, and (iii) dyspareunia prevalence/severity. Secondary outcomes included time to resumption of intercourse and sexual satisfaction. Determinants were recorded as binary/ordinal where possible; multivariable effect estimates (OR/aOR/RR) were preferred over crude associations when available. Studies on pelvic floor disorders or OASI were included when postpartum sexual outcomes were reported.

PICOS—Population: postpartum women ≤12 months; Exposure: breastfeeding; partner/family support and body image; perineal trauma/instrumentation; postpartum perineal/musculoskeletal pain; Comparison: non-exposed or alternative exposure levels; Outcomes: sexual dysfunction (instrument cut-offs), FSFI/FSFI-6/PISQ-12 scores, dyspareunia, and sexual activity resumption; Study designs: cohort, case–control, cross-sectional, and registry-based primary studies.

### 2.2. Information Sources and Search Strategy

We searched PubMed, Web of Science Core Collection, and Scopus from inception to 4 August 2025. We scanned reference lists of included studies. The gray literature and preprints were not included. The search was not updated after 4 August 2025. The search string included the following: ((“postpartum”[Title/Abstract] OR “postnatal”[Title/Abstract]) AND (sexual*[Title/Abstract] OR dyspareunia[Title/Abstract] OR “Female Sexual Function Index”[Title/Abstract] OR FSFI[Title/Abstract] OR “PISQ-12”[Title/Abstract])) AND (breastfeed*[Title/Abstract] OR lactation[Title/Abstract] OR “partner”[Title/Abstract] OR “spousal support”[Title/Abstract] OR “family support”[Title/Abstract] OR “body image”[Title/Abstract] OR perineal[Title/Abstract] OR episiotomy[Title/Abstract] OR “obstetric anal sphincter injury”[Title/Abstract] OR vacuum[Title/Abstract] OR forceps[Title/Abstract] OR “pelvic girdle pain”[Title/Abstract] OR “perineal pain”[Title/Abstract] OR musculoskeletal[Title/Abstract]).

### 2.3. Study Selection

After removing duplicates in EndNote 21, two reviewers independently screened titles and abstracts in Rayyan, followed by full-text assessment of potentially eligible reports. Disagreements were resolved by discussion until consensus was reached. The non-primary literature and articles that did not meet the eligibility criteria after full-text review were excluded. Of 1127 records initially retrieved (de-duplicated), 78 underwent full-text screening; 15 articles met inclusion for final synthesis [17,18,19,20,21,22,23,24,25,26,27,28,29,30,31]: breastfeeding and sexual function (*n* = 125 U.S. cross-sectional), partner/family support and body image (*n* = 336 Greece; 2025), postpartum dyspareunia cohorts/case–control on pain (Belgium 2017 *n* = 109; Sweden 2020 *n* = 85), large obstetric trauma/instrumentation cohorts (U.S. 2001 *n* = 615; U.S. 2008 *n* ≈ 459 analyzed at 6 months; Sweden 2024 *n* = 5328), multisite India (*n* = 3112), and additional national/regional cohorts on breastfeeding status or psychosocial factors (Poland 2023 *n* = 253; Malaysia 2023 *n* = 429; Iran 2017 *n* = 380; U.S. 2013 *n* = 160; U.S. 2019 *n* = 45; Germany 2019 *n* ≈ 330). Reasons for exclusion included absence of postpartum outcomes, qualitative-only data, or missing total *n*. Given substantial heterogeneity of metrics and timepoints, meta-analysis across all domains was not feasible; instead, we structured results by determinant domain and provided structured quantitative tables. Study counts: Breastfeeding (*n* = 5), partner/body image (*n* = 5), perineal trauma/instrumentation (*n* = 4), and postpartum pain phenotypes (*n* = 2). Adjusted estimates were available in [17,21,23,24,31]; others reported crude comparisons.

**Figure 1:**
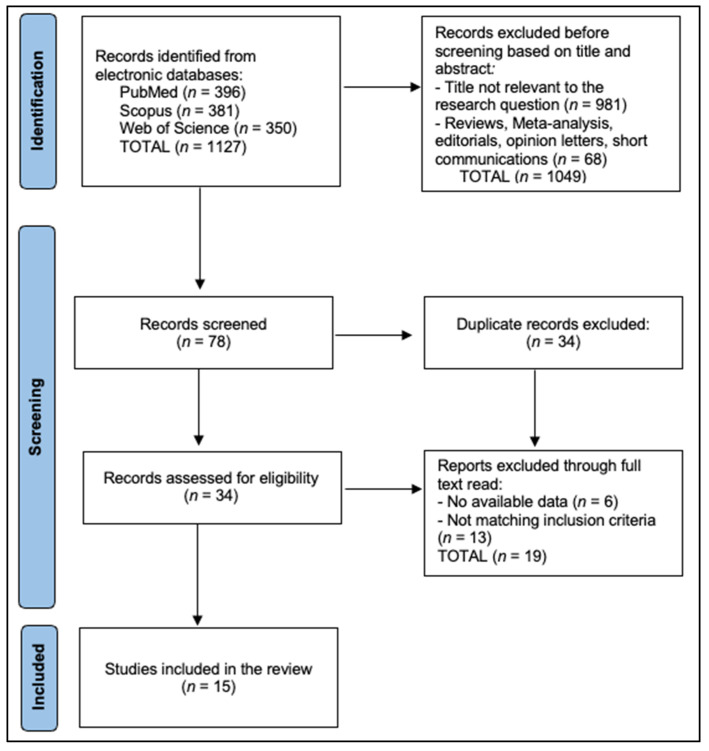
PRISMA 2020 flow diagram for study selection.

Reasons for exclusion at the full-text stage were recorded, and the study flow is summarized in the PRISMA diagram (Figure 1).

### 2.4. Data Extraction

Two reviewers independently extracted the following information for each study: study design and clinical setting, country, postpartum assessment timepoint(s), total number of participants (enrolled and analyzed), participant age and parity, definitions of each determinant, sexual function instruments and cut-offs, definitions of dyspareunia and sexual activity resumption, crude and adjusted effect estimates (with 95% confidence intervals when reported), and covariates. For studies providing only category counts or medians with IQR, we tabulated as reported. When multiple postpartum timepoints were available, we prioritized 5–6 months and 12 months; if only 3 months existed and included determinants of interest, we extracted those if within our postpartum window. Missing data cells are labeled NR (not reported). We also recorded key confounders used in models (age, parity, mode of delivery, laceration grade, depressive symptoms). All data were double-entered; discrepancies were rechecked against source PDFs or journal abstracts.

Two reviewers independently extracted study design and setting, country, postpartum assessment timepoint(s), number of participants analyzed, participant age and parity, determinant definitions, sexual function instruments (FSFI, FSFI-6, PISQ-12) and cut-offs, dyspareunia definitions, sexual activity resumption, crude and adjusted effect estimates (odds ratios, relative risks, or adjusted odds ratios, including 95% confidence intervals when these were reported in the original article), and covariates. Where studies reported 95% confidence intervals, these are presented alongside the corresponding effect estimates in the Results text and summary tables.

### 2.5. Risk of Bias Assessment

Risk of bias (RoB) for observational studies used a modified Newcastle–Ottawa Scale (NOS) with domains: Selection (0–4), Comparability (0–2), Outcome/Exposure (0–3). Total scores (0–9) were classified as Low (7–9), Moderate (5–6), and High (≤4). Two reviewers rated RoB independently with consensus resolution. Frequent concerns included sampling from single centers (selection), self-reported exposures (breastfeeding status without duration/exclusivity detail), reliance on screening cutoffs (FSFI-6 vs. FSFI full), and incomplete adjustment (for mood symptoms or relationship variables). Registry analyses (*n* > 5000) had strong selection frames but outcome self-report and possible misclassification (dyspareunia grading). Cross-sectional designs limited causal inference for psychosocial determinants (support, body image). Cohorts with repeated measures (6 weeks and 6 months) provided stronger temporal anchors. Overall, evidence quality was moderate for perineal trauma/instrumentation and postpartum pain, low-to-moderate for breastfeeding (heterogeneous timing/definitions), and low-to-moderate for partner support/body image (cross-sectional predominance).

### 2.6. Data Synthesis

Given heterogeneity in outcome timing (3/6/12 months), instruments (FSFI vs. FSFI-6 vs. PISQ-12), and exposure definitions, we used structured narrative synthesis grouping studies by determinant and timepoint, distinguishing adjusted vs. unadjusted effects. We planned a meta-analysis a priori for breastfeeding vs. sexual function, but deemed pooling inappropriate due to incompatible thresholds and reporting formats; this is reflected in GRADE downgrades for inconsistency/imprecision (Table 1).

## 3. Results

Across 15 included studies [17,18,19,20,21,22,23,24,25,26,27,28,29,30,31], designs spanned registry-based cohorts, prospective and retrospective cohorts, case–control and cross-sectional analyses, as well as one higher-level systematic review of systematic reviews and meta-analyses on postpartum couples’ sexual function [30], with postpartum assessments concentrated around 5–6 months and 12 months. Sample sizes ranged from very small (*n* = 45 in a U.S. cohort focusing on musculoskeletal pain and sexual bother [19]) to very large (*n* = 5328 in a Swedish registry cohort comparing second-degree tears vs. episiotomy [24] and *n* = 3112 in a multisite Indian cohort analyzing delivery mode and dyspareunia risk [23]). Determinant domains were distributed as follows: breastfeeding/infant-feeding ([17,20,21,25,26]), partner/family support, body image, and couple dynamics (primary cross-sectional/cohort analyses [18,31] and one secondary systematic review of systematic reviews and meta-analyses [30]), obstetric perineal trauma/instrumentation ([21,22,24]), and postpartum pain phenotypes ([19,29]). Most studies reported validated sexual outcomes—FSFI/FSFI-6 or PISQ-12—alongside dyspareunia prevalence or resumption of sexual activity; for example, FSFI was used in Germany (*n* = 330, 6-month assessment [18]) and Belgium (*n* = 109, 6 weeks and 6 months [20]), while PISQ-12 quantified function at 6 months in the U.S. Pelvic Floor Disorders Network cohort [22], as presented in Table 2.

Breastfeeding clustered with lower sexual function scores and higher dyspareunia odds at early postpartum timepoints, with attenuation by 6–12 months in some studies. In a U.S. 5–6-month cohort (*n* = 125), median FSFI was lower among primarily breastfeeding vs. formula-feeding women (20.8 [IQR 10–24] vs. 24.5 [19.5–27.8]; *p* = 0.009), with multivariable models also implicating perineal laceration, progestin LARC, and single status [17]. A Belgian prospective cohort found breastfeeding associated with dyspareunia at 6 weeks (*p* = 0.045), but not at 6 months; primiparity remained associated at 6 months [20]. In classic U.S. data, breastfeeding conferred ≥4-fold higher odds of dyspareunia at 6 months (OR 4.4, 95% CI 2.7–7.0) [21]. An Iranian cross-sectional study reported exclusive breastfeeding independently predicted sexual dysfunction (aOR 2.47, 95% CI 1.21–5.03) with additional risk from primiparity [25]. Polish survey data similarly linked breastfeeding to lower sexual function indices, though detailed quantitation was NR in the abstract [26]. Overall, directionally consistent associations were strongest at earlier postpartum timepoints and when exposure was defined as exclusive or current breastfeeding [17,20,21,25,26], as presented in Table 3.

**Figure 2:**
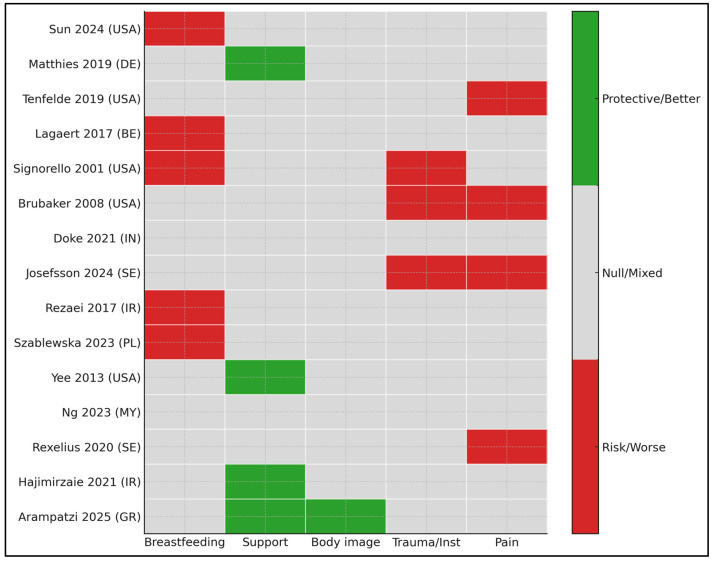
Evidence heatmap (direction of association by determinant and study) [17,18,19,20,21,22,23,24,25,26,27,28,29,30,31].

Across the 15 included studies [17,18,19,20,21,22,23,24,25,26,27,28,29,30,31], perineal trauma or instrumentation and postpartum pain were consistently associated with worse sexual outcomes, whereas higher levels of partner and family support and more positive body image were associated with better sexual function. Breastfeeding is frequently linked to lower early postpartum function or dyspareunia, although findings are not uniform across all cohorts and timepoints. The pattern highlights actionable clinical levers—early pain/perineal complication management and partner/body image-oriented support—while emphasizing context-sensitive counseling for lactating women (Figure 2).

Higher partner/family support and more positive body image correlated with better sexual function across culturally diverse samples. In Greece (*n* = 336), higher support and favorable body image scores were associated with higher FSFI total and domain scores after multivariable adjustment, while low support/negative body image aligned with dysfunction [31]. A German cohort (*n* = 330) observed that better partnership quality tracked with higher FSFI at 6 months postpartum; models also considered breastfeeding and LARC use [18]. A systematic review of systematic reviews and meta-analyses evaluating postpartum couples’ sexual function within a biopsychosocial framework similarly found consistent correlations between women’s FSFI scores and couple-level relational and psychosocial indices [30]. In Malaysia (*n* = 429), lower coital frequency independently predicted FSFI-6-defined dysfunction, highlighting behaviorally mediated pathways [28]. U.S. prospective data at 12 months indicated psychosocial predictors (including mood and relationship factors) shaped sexual activity resumption and problems, complementing partner–support findings [27]. Across studies that captured sleep/fatigue indicators, lower energy or poor sleep was descriptively associated with lower FSFI desire/arousal and higher sexual distress, though measures were heterogeneous and often unadjusted. Collectively, these studies support psychosocial context as a modifiable determinant of postpartum sexual function beyond somatic recovery [18,27,28,30,31], as described in Table 4.

In primiparous U.S. data, dyspareunia prevalence was 41% at 3 months and 22% at 6 months; vacuum or forceps delivery increased 6-month dyspareunia odds (OR 2.5, 95% CI 1.3–4.8), and breastfeeding raised dyspareunia odds (OR 4.4, 95% CI 2.7–7.0) [21]. In the Pelvic Floor Disorders Network cohort at 6 months, 36% of sexually active women reported pain during sex, the mean PISQ-12 was 39 ± 4, and 88–94% had resumed sexual activity, depending on exposure; sexual activity was lower after OASI [22]. The Swedish registry study reported ~30–35% mild/moderate and 2–4% strong dyspareunia at 12 months; pain at 8 weeks predicted strong dyspareunia (aOR ~4.0), and episiotomy increased dissatisfaction (aOR 2.4) [24]. In India, dyspareunia was ~50% at 6 weeks after vaginal birth and ~33% at 6 months; CS was linked to higher non-resumption at 6 weeks (RR 1.14), with dyspareunia risk lower after CS at both 6 weeks (RR 0.59) and 6 months (RR 0.49) [23]. A Swedish case–control study found persistent pelvic girdle pain associated with more pain during intercourse (*p* < 0.001), higher sexual avoidance (*p* < 0.001), and worse sexuality scores with increasing depressive symptoms (regression β −0.41 per depression unit) [29]. Overall, early perineal pain, higher trauma grades/OASI, and assisted vaginal delivery signal elevated dyspareunia risk trajectories [21,22,23,24,29], as presented in Table 5.

**Figure 3:**
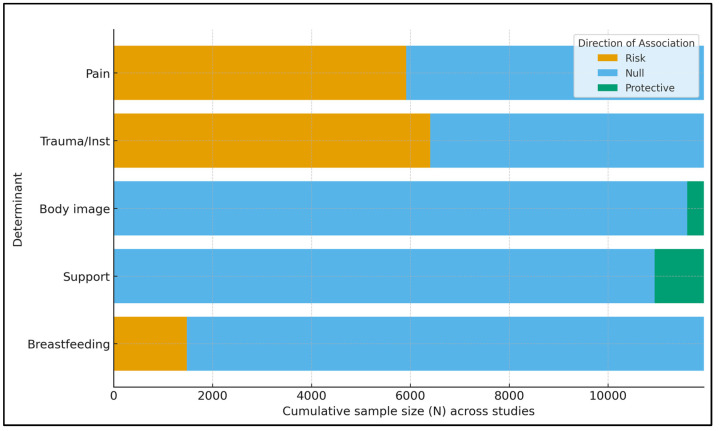
Weighted evidence balance by determinant (cumulative N).

The N-weighted evidence balance shows that studies indicating risk associations with postpartum sexual outcomes accumulate the largest sample sizes for perineal trauma/instrumentation and postpartum pain, underscoring these as high-priority clinical targets. In contrast, partner/family support and positive body image contribute the bulk of the protective sample size, albeit across fewer total participants than the trauma/pain domains. For breastfeeding, the cumulative bar reflects a meaningful risk-leaning component but also substantial null/mixed sample size, consistent with heterogeneity by timing and context. Because these bars are weighted by sample size rather than study quality, they should be interpreted alongside risk of bias assessments (Figure 3).

## 4. Discussion

### 4.1. Summary of Evidence

Our synthesis that breastfeeding is associated with lower FSFI scores and higher dyspareunia odds in early–mid puerperium aligns with population data showing lubrication problems and pain are common at 6–12 months postpartum, particularly among lactating primiparae [32]. Cross-sectional evidence at ~3 months corroborates a gradient by infant-feeding method, with exclusive breastfeeding linked to higher sexual dysfunction prevalence and lower domain scores (desire, arousal, lubrication, orgasm, and pain) than formula feeding [33]. A 2025 meta-analysis further reports slightly lower pooled FSFI scores in exclusively breastfeeding women versus complementary feeding, while emphasizing that sexual difficulties are frequent regardless of feeding type, underscoring the need to couple lactation support with sexuality counseling rather than viewing them as competing priorities [34]. Prior syntheses emphasize high PSD burden but seldom integrate somatic and psychosocial determinants within the same framework or standardize time windows. Our review adds disaggregated patterns by early pain/perineal morbidity and makes explicit the protective roles of partner support/body image, while aligning with large cohort trajectories showing improvement by 12 months.

We report adjusted estimates preferentially and indicate where only crude associations were available. Ranges across studies: dyspareunia 6 weeks ~50% after vaginal birth, decreasing to ~22–35% by 6–12 months; breastfeeding-associated dyspareunia at 6 months OR ~4.4 in one large cohort; assisted vaginal birth OR ~2.5 for 6-month dyspareunia [35]. Contemporary obstetric quality initiatives emphasize prevention, recognition, and optimal repair of perineal trauma as the actionable levers for sexual outcomes [36]. Urogynecologic cohorts likewise document worse sexual function after OASI at 6–12 months, with persistent pain and reduced activity compared with women without sphincter injury [32]. Nuanced obstetric analyses indicate that episiotomy does not uniformly protect sexual comfort and, in some contexts, relates to higher dissatisfaction versus spontaneous second-degree tears [37]. In contrast, large prospective cohorts suggest that by 12 months, sexual function often converges toward baseline irrespective of delivery mode, reinforcing that early differences reflect transient recovery and trauma burden rather than permanent mode effects [38].

Our results on postpartum pain phenotypes—perineal pain and PGP—resonate with broader evidence linking musculoskeletal/pelvic pain to avoidance, reduced arousal, and dyspareunia. Reviews of risk factors repeatedly identify perineal trauma severity and lactation as somatic contributors, with mood symptoms and fatigue compounding pain–sex interactions [39]. Importantly, targeted rehabilitation appears promising: pelvic floor rehabilitation protocols (PFMT, biofeedback, manual therapy, and device-assisted training) have shown improvements in dyspareunia and sexual function in controlled and observational studies, including postpartum samples [40,41,42]. While high-quality RCT data focused exclusively on postpartum dyspareunia remain limited, early comparative work suggests vibrating vaginal cones may improve dyspareunia and sexual function similarly or better than PFMT alone in clinical practice cohorts [41], supporting your call for multidisciplinary aftercare pathways that integrate pelvic health PT with pain-science education.

The psychosocial layer in our review—partner support and body image—finds convergent support in emerging perinatal couples research. A daily diary study of new-parent couples showed that sexual satisfaction mediates the association between body satisfaction and relationship satisfaction, highlighting sexual function as a proximal mechanism through which body image influences couple adjustment in early postpartum months [43]. Conceptual and empirical work focused specifically on postpartum women also links more positive body image with greater comfort in partnered sexual activities and better sexual functioning, suggesting body image interventions as plausible adjuncts to somatic care [43]. These findings dovetail with our multivariable signals for support and body image constructs on FSFI outcomes, arguing for routine screening and brief, scalable interventions (cognitive–behavioral, self-compassion-based, and partner-inclusive strategies) alongside physical recovery. Chronic fatigue and sleep disruption plausibly depress desire/arousal via reduced physiological readiness and motivational bandwidth. Observational postpartum work identifies fatigue among recurrent correlates of sexual problems alongside mood and body image [40]; however, validated fatigue scales were seldom incorporated into multivariable models in the included studies. Future cohorts should prospectively measure fatigue/sleep with standardized tools and report domain-specific FSFI results.

Consistent signals with the greatest cumulative N were observed for perineal trauma/instrumentation and early pain (moderate certainty). Breastfeeding effects were most apparent before ~6 months and attenuated thereafter (low–moderate certainty), contingent on exclusivity and timing. Evidence for partner support and body image was positive but predominantly cross-sectional (low–moderate certainty). Key gaps include standardized outcome timing, postpartum-validated FSFI thresholds, and fatigue/sleep covariates.

Targeted pelvic health rehabilitation typically includes pelvic floor muscle training (PFMT) with home practice, biofeedback-guided training, manual therapy for myofascial/perineal scar pain, and graded dilator or vibrator programs; postpartum cohorts and clinical series suggest symptom improvements, including dyspareunia, with PFMT and vibration cones [41,42]. Evidence for neuromuscular electrical stimulation specifically in postpartum dyspareunia is limited; where used, it should be individualized and adjunctive.

Abstinence/confinement norms, access to perineal repair quality, and postpartum counseling vary widely; LMIC surveys show earlier resumption driven by partner expectations and economic pressures [6,7,28]. Generalizability from single-center high-income cohorts is therefore limited and underscores the need for culturally adapted counseling and community-delivered pelvic health support.

### 4.2. Limitations

The evidence base is heterogeneous in design, timing (3, 6, and 12 months), and outcome measurement (FSFI vs. FSFI-6; varied dyspareunia definitions). Many exposures and outcomes were self-reported, breastfeeding was inconsistently specified for duration/exclusivity, and psychosocial constructs used diverse, sometimes non-validated scales. We did not search gray literature/preprints, as it might introduce potential publication bias. Although we did not impose language limits in PubMed, the requirement for PubMed indexing may introduce language bias. Quantitative pooling of breastfeeding studies was considered but not performed due to incompatible thresholds and timepoints; future reviews could attempt random-effects meta-analysis after harmonizing definitions. Cross-sectional predominance limits causal inference; residual confounding by mood, relationship quality, contraception, and cultural norms is likely. We did not calculate inter-rater reliability a priori—this is noted as a limitation. Registry data improved sampling but still relied on self-report for pain and sexual outcomes. Between-study variability precluded meta-analysis across all domains, and generalizability may be constrained by single-center settings and underrepresentation of low-resource contexts and multipartner perspectives.

## 5. Conclusions

Perineal trauma and early pain were the most consistent risk factors for postpartum sexual pain and dysfunction (moderate certainty). Breastfeeding-related symptoms showed early, context-dependent associations that attenuated by 12 months (low–moderate certainty). Partner support and positive body image were protective but supported largely by cross-sectional data (low–moderate certainty). These findings support integrated postpartum care models, yet confirmatory longitudinal and intervention studies, with standardized instruments and timepoints, are needed to establish causal pathways.

## Figures and Tables

**Table 1:** Summary of Findings (GRADE) for main determinants and postpartum sexual outcomes (≤12 months).

Determinant	Outcome	Effect (Direction) *	Certainty (GRADE)	Key Reasons for Rating
Perineal trauma/instrumentation	Dyspareunia; sexual activity; sexual function (PISQ-12/FSFI)	Higher risk of dyspareunia; lower sexual activity and sexual function	Moderate	Observational designs; consistent direction of effect; imprecision in exact magnitude; heterogeneity in timing and outcome measures
Early perineal or pelvic pain	Dyspareunia; sexual avoidance/distress	Higher risk of dyspareunia and sexual avoidance or distress	Moderate	Observational designs; consistent association between early pain and later sexual symptoms; some residual confounding
Breastfeeding (especially exclusive breastfeeding in early months)	Dyspareunia; FSFI total and domain scores	Higher risk of dyspareunia and lower FSFI scores in early postpartum, with attenuation by 12 months	Low–Moderate	Inconsistency across timepoints; heterogeneity in breastfeeding definitions; imprecision; predominantly observational data
Partner and family support; positive body image	FSFI total/domains; sexual distress	Better sexual function (higher FSFI) and lower sexual distress	Low–Moderate	Predominantly cross-sectional designs; potential residual confounding; consistent direction of association across settings
Postpartum pelvic girdle pain and other musculoskeletal pain	Dyspareunia; global sexual function	Higher sexual pain, greater sexual avoidance, and poorer global sexual function	Moderate	Case–control and cohort designs; consistent association between pain and worse sexual outcomes; some unmeasured confounding

* Narrative direction of effect based on non-poolable observational data; no meta-analysis was performed.

**Table 2:** Study characteristics and determinants.

#	Study (Year, Country)	Design and Postpartum Timepoint	Determinant(s)	Sexual Outcome(s)	Total *n*
[17]	Sun et al., 2024 (USA)	Cross-sectional; 5–6 months	Breastfeeding vs. formula; contraception; perineal laceration	FSFI total, DIVA	125
[18]	Matthies et al., 2019 (Germany)	Cohort; 6 months	Partner relationship quality; breastfeeding	FSFI	330 (enrolled prenatally)
[19]	Tenfelde et al., 2019 (USA)	Cohort; 5–6 months	Musculoskeletal pain; depressive symptoms	Sex activity and bother	45
[20]	Lagaert et al., 2017 (Belgium)	Prospective cohort; 6 weeks and 6 months	Breastfeeding; parity	FSFI; dyspareunia prevalence	109
[21]	Signorello et al., 2001 (USA)	Retrospective cohort; 3 and 6 months	Perineal trauma grade; instrumentation; breastfeeding	Dyspareunia; orgasm; satisfaction	615
[22]	Brubaker et al., 2008 (USA)	Cohort (CAPS); 6 months	OASI vs. none; delivery mode	PISQ-12; sexual activity; pain	459 sexually active at 6 mo
[23]	Doke et al., 2021 (India)	Multisite cohort; 6 weeks and 6 months	Cesarean vs. vaginal; perineal injury; rural residence	Non-resumption; dyspareunia (RR)	3112
[24]	Josefsson et al., 2024 (Sweden)	Registry cohort; 8 weeks and 12 months	2nd-degree tear vs. episiotomy; infection; re-suturing; pain	Dyspareunia; satisfaction	5328
[25]	Rezaei et al., 2017 (Iran)	Cross-sectional; 8 weeks–8 months	Exclusive breastfeeding; parity	FSFI dysfunction (cut-off)	380
[26]	Szablewska et al., 2023 (Poland)	Cross-sectional; ≤1 year	Breastfeeding practices and sexuality	Sexual life self-report	253
[27]	Yee et al., 2013 (USA)	Prospective; 12 months	Predictors (delivery, mood, etc.)	Sexual activity; problems	160
[28]	Ng et al., 2023 (Malaysia)	Cross-sectional; 6 months	Sociodemographic; intercourse frequency	FSFI-6 dysfunction	429 analyzed
[29]	Rexelius et al., 2020 (Sweden)	Case–control; postpartum persistent PGP	Pelvic girdle pain vs. healthy	McCoy Sexuality; dyspareunia	85 (46 + 39)
[30]	Hajimirzaie et al., 2025 (Iran)	Systematic review	Couple dynamics and sexuality	FSFI (women), IIEF (men), multiple scales	NR
[31]	Arampatzi et al., 2025 (Greece)	Cross-sectional; ≤1 year	Spousal/family support; body image; lifestyle	FSFI total/domains	336

Abbreviations: FSFI, Female Sexual Function Index; FSFI-6, 6-item FSFI; IIEF, International Index of Erectile Function; PGP, pelvic girdle pain; PISQ-12, Pelvic Organ Prolapse/Urinary Incontinence Sexual Questionnaire-12; DIVA, Day-to-Day Impact of Vaginal Aging; NR, not reported; OASI, obstetric anal sphincter injury; #, reference number.

**Table 3:** Breastfeeding/infant-feeding and postpartum sexual function.

Study	Timepoint	Exposure Definition	Sexual Outcome	Key Findings
Sun 2024 (USA) [17]	5–6 months	Self-reported primarily breastfeeding vs. formula	FSFI total (median, IQR)	20.8 (IQR 10–24) BF vs. 24.5 (19.5–27.8) FF; *p* = 0.009; lower FSFI associated with BF, perineal laceration, progestin LARC, single status (multivariable)
Lagaert 2017 (Belgium) [20]	6 weeks and 6 months	Breastfeeding yes/no	Dyspareunia; FSFI	Dyspareunia assoc. with BF at 6 wks (*p* = 0.045); association not significant at 6 mo; primiparity remained associated at 6 mo
Signorello 2001 (USA) [21]	6 months	Breastfeeding yes/no	Dyspareunia	Breastfeeding ≥4× odds of dyspareunia at 6 mo (OR 4.4; 95% CI 2.7–7.0)
Rezaei 2017 (Iran) [25]	8 weeks–8 months	Exclusive vs. non-exclusive BF	FSFI dysfunction	Exclusive BF aOR 2.47 (1.21–5.03) for dysfunction; primiparity aOR 1.78
Szablewska 2023 (Poland) [26]	≤12 months	Breastfeeding status	Sexual life self-report	BF associated with lower sexual function measures (narrative; numeric detail NR in abstract)

Abbreviations: aOR, adjusted odds ratio; BF, breastfeeding; FF, formula feeding; FSFI, Female Sexual Function Index; IQR, interquartile range; LARC, long-acting reversible contraception; NR, not reported; OR, odds ratio.

**Table 4:** Partner/family support and body image, and postpartum sexual function.

Study	Construct	Instrument (If Any)	Sexual Outcome	Key Finding
Arampatzi 2025 (Greece) [31]	Spousal/family support; body image; lifestyle	FSFI; support/body image scales	FSFI total/domains	Higher support and positive body image → better FSFI; low support/negative body image associated with dysfunction (multivariable)
Matthies 2019 (Germany) [18]	Partner relationship quality	FSFI	FSFI	Better partnership quality associated with higher FSFI; breastfeeding and LARC also modeled
Hajimirzaie 2025 (Iran) [30]	Couple dynamics	biopsychosocial model (systematic review/secondary synthesis)	biopsychosocial model (systematic review/secondary synthesis)	Relational (biopsychosocial) factors consistently influence postpartum sexual function in both partners, supporting couple-centered care.
Ng 2023 (Malaysia) [28]	Frequency of intercourse; sociodemographics	FSFI-6	FSFI-6 dysfunction	Lower coital frequency strongly associated with FSD (multivariable); cultural context discussed
Yee 2013 (USA) [27]	Predictors (mood, delivery, etc.)	Structured survey	Sexual activity/problems	Psychosocial factors predicted sexual activity resumption at 12 months

Abbreviations: FSFI, Female Sexual Function Index; FSFI-6, 6-item FSFI; LARC, long-acting reversible contraception.

**Table 5:** Perineal trauma/instrumentation and postpartum pain (pelvic/perineal) vs. sexual outcomes.

Study	Time	Exposure(s)	Dyspareunia %	FSFI Total	Resumed Sex by X	Other Numeric Effect
Signorello 2001 (USA) [21]	3 and 6 months	2nd-degree; 3rd/4th; instrumentation; BF	41% (3 months); 22% (6 months)	NR	NR	Vacuum/forceps OR 2.5 for dyspareunia at 6 months; BF OR 4.4
Brubaker 2008 (USA) [22]	6 months	OASI vs. none; mode	Pain during sex 36% of sexually active individuals	PISQ-12 mean 39 ± 4	88–94% sexually active by 6 months (varied by group)	Activity lower after OASI
Josefsson 2024 (Sweden) [24]	12 months	2nd-degree vs. episiotomy; infection; re-suturing; pain at 8 wks	~30–35% mild/moderate; 2–4% strong	NR	83–85% had intercourse last 3 months	Pain at 8 weeks aOR ~4.0 for strong dyspareunia; episiotomy ↑ dissatisfaction (aOR 2.4)
Doke 2021 (India) [23]	6 weeks and 6 months	Cesarean vs. vaginal; perineal injury	~50% dyspareunia at 6 weeks (vaginal); ~33% at 6 months (vaginal)	NR	Non-resumption higher after CS at 6 weeks (RR 1.14)	Dyspareunia RR after CS 0.59 (6 weeks) and 0.49 (6 months)
Rexelius 2020 (Sweden) [29]	Postpartum (months)	Persistent pelvic girdle pain vs. healthy	Higher pain during intercourse (*p* < 0.001)	McCoy total: group diff NR; regression β −0.41 per depression unit	NR	Sexual avoidance ↑ (*p* < 0.001)

Abbreviations: aOR, adjusted odds ratio; CS, cesarean section; OASI, obstetric anal sphincter injury; OR, odds ratio; PISQ-12, Pelvic Organ Prolapse/Urinary Incontinence Sexual Questionnaire-12; RR, risk ratio.

## Data Availability

No new data were created or analyzed in this study. Data sharing is not applicable to this article.

## Supplementary Materials

The following supporting information can be downloaded at: https://www.mdpi.com/article/10.3390/healthcare13222977/s1, PRISMA checklist [16].
